# Stage-specific modulation of *Drosophila* gene expression with muscle GAL4 promoters

**DOI:** 10.1080/19336934.2024.2447617

**Published:** 2025-01-07

**Authors:** Ziwei Zhao, Erika R Geisbrecht

**Affiliations:** Department of Biochemistry and Molecular Biophysics, Kansas State University, Manhattan, KS, USA

**Keywords:** Muscle, promoter, *Drosophila*, GAL4/UAS, expression

## Abstract

The bipartite GAL4/UAS system is the most widely used method for targeted gene expression in *Drosophila melanogaster* and facilitates rapid *in vivo* genetic experimentation. Defining precise gene expression patterns for tissues and/or cell types under GAL4 control will continue to evolve to suit experimental needs. However, the precise spatial and temporal expression patterns for some commonly used muscle tissue promoters are still unclear. This missing information limits the precise timing of experiments during development. Here, we focus on three muscle-enriched GAL4 drivers (*Mef2*-GAL4, *C57*-GAL4 and *G7*-GAL4) to better inform selection of the most appropriate muscle promoter for experimental needs. Specifically, *C57*-GAL4 and *G7*-GAL4 turn on in the first or second instar larval stages, respectively, and can be used to bypass myogenesis for studies of muscle function after development.

## Introduction

*Drosophila melanogaster* is an excellent experimental model for *in vivo* genetics experiments due to its highly conserved protein sequences, short growth cycles, ease of laboratory culture, and well-established genetic expression systems [1]. Most laboratories employing *Drosophila* as a model use and benefit from the GAL4/UAS system to knockdown gene expression, rescue loss-of-function mutations, and express mutated proteins, namely human disease alleles [2]. These tools together make *Drosophila* an unparalleled model in understanding gene function.

In 1988, the Ptashne lab showed that the *Saccharomyces cerevisiae* yeast GAL4 protein can stimulate transcription of reporter genes in multiple cell types, including mammalian cells, plant cells, and *Drosophila* larvae [3–5]. In 1993, Andrea Bread and Norbert Perrimon extended this GAL4/UAS technique to manipulate *Drosophila* gene expression in a cell- or tissue-specific manner [6]. This bipartite approach makes use of the transcription factor GAL4 and a ‘responder’ element under the control of a cis-acting regulatory sequence called the upstream activating sequence (UAS). The binding of GAL4 to these engineered UAS sites activates transcription of downstream components, which may include expression of wild-type or mutated genes, RNAi sequences, and reporter constructs, among others [2,7]. Implementation of this technique is still widely utilized to study the impact of genes during *Drosophila* development and growth.

With the need to regulate gene expression in independent tissues and at different stages, numerous tissue- and/or cell-specific promoters have been constructed to allow for precise gene control. For example, collections of GAL4 drivers exist for targeting specific cell types in the *Drosophila* intestine [8] or to manipulate specific motor neuron types, such as the glutamatergic motor neurons Ib or Is [9]. Alternatively, the development of Split-GAL4 allows for single-cell resolution based upon the overlapping expression of two independent enhancers [10]. Additional temporal control of gene expression may be necessary if alteration of gene function (e.g. overexpression, dominant-negative, RNAi knockdown) causes lethality or altered phenotypes prior to the desired stage of analysis. While methods such as temperature-sensitive GAL80 (GAL80ts), tetracycline-inducible GAL80 (Tet-off GAL80), Gene-switch, and auxin-inducible gene expression systems (AGES) have been developed [11–14], these expression systems have limitations and may complicate experimental design. Thus, it is valuable to continue developing new and further characterizing existing GAL4 lines that can be used for more precise spatial and temporal control.

The focus of this paper is to characterize the developmental gene expression patterns of three muscle driver lines: *Mef2*-GAL4, *C57*-GAL4, and *G7*-GAL4. Myocyte enhancer factor-2 (*Mef2*) is one of the earliest identified and well-studied transcription factors in both *Drosophila* and mammalian muscles [15,16]. Since *Drosophila* Mef2 protein is present in the developing mesoderm and persists throughout somatic, visceral, and cardiac cells spanning embryogenesis into adulthood [17], the *Mef2*-GAL4 driver provides a well-characterized control to compare muscle expression during development. However, there is lack of primary data describing the exact timing of target gene expression by the widely used *C57*-GAL4 and *G7*-GAL4 muscle promoters [18–28]. *C57*-GAL4 was first published as *BG57*-GAL4 and expression was reported in all larval muscles from the first instar (1^st^) to the third (3^rd^) instar stage as well as two sensory cell bodies in the body wall and in mesodermally derived gut tissues [18]. However, unpublished reports suggested possible expression in late embryogenesis. In 2008, while *G7*-GAL4 was described as having expression in all muscles beginning from the second instar larval stage [27], the supporting references did not include primary data to support this conclusion. To clarify these discrepancies, here we pinpoint the spatial and temporal expression of these muscle GAL4 drivers using UAS-mCherry.NLS (mCh) as a readout.

## Materials and methods

### *Drosophila* stocks and husbandry

All stocks were reared at 25°C on standard cornmeal-molasses-yeast media. Stocks obtained from the Bloomington *Drosophila* Stock Center (BDSC) are indicated with BL followed by the stock number. The following GAL4 drivers were used to direct expression of the UAS-mCherry.NLS (BL38424; FBti0147460) responder line in muscle tissue: *Mef2*-GAL4 (BL27390; FBti0115746), *C57*-GAL4 (FBti0016293) (L. Wallrath), and *G7*-GAL4 (M. Baylies). Note that at the time of publication, two commercially available stocks containing *C57*-GAL4 (BL32556 and BL52001) are also available at the BDSC.

### Visualization of muscle tissue expression

#### Embryo fixation and imaging

Crosses of the desired genotypes were set up in FlyStuff embryo collection cages (#59–105; Genesee Scientific, San Diego, CA) and inverted onto 35 mm petri dishes containing apple juice-agar with fresh yeast paste. The pots were placed in an incubator at a defined temperature (25°C) and humidity (60%) where flies were allowed to lay eggs. After 2–3 hrs, the collection plate was further staged for ~15 hrs and a new plate was used to collect the next batch of embryos. Using a wet paintbrush, the embryos were collected into a mesh basket filled with 1× PBS and rinsed to remove yeast paste. Embryos were dechorionated in freshly diluted 50% bleach and rinsed with a solution of 0.7% NaCl/0.04% Triton X-100 before transferring to small glass vial containing a 1:1 fixative solution of heptane and 4% formaldehyde in PEM (0.1 M Pipes, pH 8.0; 2 mm MgSO_4_; 1 mm EGTA). The embryos were shaken vigorously for 12 minutes on a platform shaker to ensure penetration of the fixative solution. The bottom layer of fixative was removed, and an equal volume of methanol was added. The settled embryos were transferred to a microcentrifuge tube, washed three times with methanol, followed by three washes with PBT. Embryos were blocked in PBT with 5% normal goat serum for 30 min before incubating in rat anti-Tropomyosin (Tm) MAC141 (1:50; Babraham Institute, Cambridge, UK) in PBT and 5% normal goat serum at 4°C overnight or at room temperature for 2 hours. Embryos were washed three times for 10 minutes each in PBT and reblocked in PBT with 5% normal goat serum for 30 minutes. Fluorescent anti-rat Alexa Fluor 488 (1:400, ThermoFisher Scientific, Waltham, MA) and Hochest (1:400, Invitrogen, Waltham, MA) were added for 1 hour at room temperature. The embryos were again washed three times for 10 minutes each in PBT. After washing, the embryos were mounted onto glass slides in anti-fade mounting medium. Samples were imaged on an LSM 700 laser scanning confocal microscope using a ZeissPlan-Apochromat 20×/0.8 M27 objective at 0.5× zoom. 1024 × 1024 pixel images were acquired using PMT detectors. For acquisition of the signal corresponding to TM, samples were illuminated with a 488 nm laser using the following parameters: 1.06 AU (pinhole size), 2.0% (laser power), 650 (gain) and 0 (offset). mCh signal was detected using a 555 nm laser with the following parameters: 0.94 AU (pinhole size), 2.0% (laser power), 850 (gain) and 0 (offset). Nuclei were imaged with a 405 nm laser using the following parameters: 1.26 AU (pinhole size), 2.0% (laser power), 680 (gain) and 0 (offset). Line averaging of 4 was applied to all channels. Z-stack images were acquired with a total of 10 slices corresponding to a 38.4 µm z-step size. Image processing was performed using ImageJ (NIH) and assembled into figures with Adobe Photoshop.

#### Live larval muscle imaging

The desired larval stages were collected on apple juice plates as described above. Embryo plates were kept at 25°C for 24 hr intervals to collect similarly staged larvae at the 1^st^ instar (24–48 hrs AEL), 2^nd^ instar (48–72 hrs AEL), and wandering 3^rd^ instar stages (112–120 hrs AEL). Live larvae were transferred using a needle probe, placed onto a Sylgard plate, and put at −20°C for 10 mins to slow down muscle movements. Larvae were then transferred onto a glass slide and secured with clear nail polish. A coverslip was added with small pieces of clay at the corners for support. As above, images were taken on a Zeiss LSM 700 confocal microscope using Zeiss ZEN Black software. All published images were taken identical to above using either 20× (1st instar; gain = 670) or 40× (2^nd^ instar; gain = 650 and 3^rd^ instar; gain = 600) objectives at 0.5× zoom with laser power set at 2.0% for each channel. Gain settings for the 555 nm laser detecting mCh signal = 850. Image processing was performed using ImageJ (NIH) an assembled into figures with Adobe Photoshop.

### mCh protein level determination

#### Embryo sample preparation

Embryo samples were homogenized in 50 μl lysis buffer (50 mm Tris-HCl pH 7.5, 150 mm NaCl, 1% Triton X-100, 10% glycerol) plus inhibitors (1× Halt protease inhibitor cocktail, 1× Halt phosphatase inhibitor cocktail, 1 mm Na_3_VO_4_, 1 mm NaF, 1 mm PMSF, 10 µM MG132, 0.001% PTU, 5 mm NPPS). Bicinchoninic acid assay (BCA) was performed on embryo lysates (Pierce BCA Protein Assay Kit 23,225; ThermoFisher) and 5 μg of total protein was loaded per lane.

#### Larval sample preparation

Dissected muscle carcasses were placed into 3× SDS sample buffer [188 mm Tris-HCl (pH 6.8), 3% (w/v) SDS, 30% (v/v) glycerol, 0.01% (w/v) bromophenol-blue, and 15% (v/v) β-mercaptoethanol], boiled at 95°C for 3 min, homogenized, boiled for an additional 10 min at 95°C, and centrifuged at 20,000 × g for 1 min to pellet debris. 10 ul of larval lysates were loaded per lane.

#### Western blotting

The resulting protein samples were separated by sodium dodecyl sulphate polyacrylamide gel electrophoresis (SDS-PAGE) and transferred to the nitrocellulose blotting membrane (pore size 0.45 μm, Cytiva, Marlborough, MA) using the Trans-Blot® TurboTM Transfer System (Bio-Rad, Hercules, CA). Membranes were probed with the following primary antibodies: rabbit anti-mCherry ab167453 (1:1000, Abcam, Waltham, MA) mouse anti-ATP5A ab14748 (1:10000, Abcam, Waltham, MA). IRDye 800CW goat anti-mouse and goat anti-rabbit secondary antibodies (LI-COR Biosciences, Lincoln, NE) were used at 1:10000 and anti-ATP5α was used as a loading control. Membranes were developed using the LI-COR Odyssey XF and quantitation of relative protein levels was performed in Empiria Studio Software (LI-COR Biosciences, Lincoln, NE).

## Results & discussion

### Only Mef2-GAL4 is detected in stage 13 to 16 embryos

**Figure 1.**
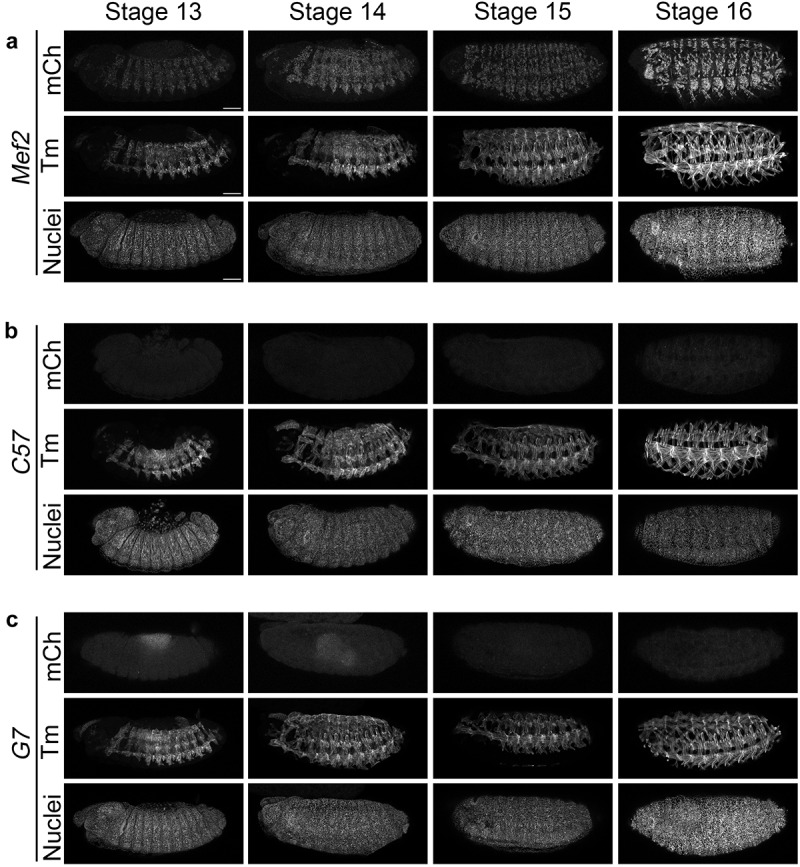
Only the *Mef2* promoter expresses mCh during embryonic muscle development. (a-c) Maximum intensity projections of embryos at stages 13–16 with *Mef2*-GAL4 (a), *C57*-GAL4 (b), or *G7*-GAL4 (c). UAS-mCherry.Nls (mCh) is the responder line to assess the timing of gene expression. Tropomyosin (Tm) staining reveals the somatic muscle pattern at each stage of development and Hoechst stain labels nuclei. While *Mef2*-GAL4 promotes expression at all stages of myogenesis, there is no visible signal with the *C57*-GAL4 or *G7*-GAL4 drivers. Scale bar: 50 μm.

The well characterized *Mef2*-GAL4 driver promotes expression in embryonic mesodermal cells from stage 7 onwards and subsequently in the cardiac, visceral, and somatic musculature [29]. The goal of this study is to clarify the exact stage at which *C57*-GAL4 or *G7*-GAL4 turn on compared to *Mef2*-GAL4 in terms of target gene expression during development. Even though Mef2 is expressed earlier, we only documented embryos at key stages in myogenesis: stage 13 (myoblast fusion ongoing), stage 14 (the peak of myoblast fusion), stage 15 (individual muscle fibres are recognizable), and stage 16 (mature muscle pattern completed) [30,31]. Tropomyosin (Tm) was used to stain the developing body wall muscles and to confirm staging by assessing development of the midgut. Consistent with published literature [29] and summarized in Table 1, the *Mef2*-GAL4 driver robustly expressed mCh from stage 13 to stage 16 (Figure 1a). This fluorescence signal intensified during muscle development as fusing muscles increased the number of mCh-expressing nuclei. We could not detect *C57*-GAL4 (Figure 1b) or *G7*-GAL4 (Figure 1c) mCh expression in embryo stages 13 to 16 using this approach. In summary, *Mef2*-GAL4, but not *C57*-GAL4 or *G7*-GAL4, initiate target gene expression during embryogenesis.

**Table 1.** Summary of GAL expression patterns compiled from the literature and data in this paper.

	*Mef2*-GAL4	*C57*-GAL4	*G7*-GAL4
Stages of somatic muscle expression	Embryo – adult	1^st^ − 3^rd^ instar larvae	2^nd^ − 3^rd^ instar larvae
Reported expression pattern	Mesoderm expression at stage 7 onwards, then expressed in somatic muscle, visceral muscle and cardiac cells. [29]	All larval body wall muscles, two sensory cell bodies in the body wall, and mesodermal-derived tissues, including the gut.[18]	Expression in all muscles beginning from the second instar larval stage. [27]
Expression pattern using mCh reporter (this study)	We only documented stage 13–16 expression in the somatic muscle, visceral muscle and cardiac cells.	All larval body wall muscles, strong expression in salivary gland, and weak midgut expression.	All larval body wall muscles, weak expression in salivary gland
Gene expression and/or gene insertion	Mef2	Likely inserted into the upstream coding region of neuromusculin (80C2-80C4) based on plasmid rescue experiments (personal communication from Ulrich Thomas)	Possibly inserted into U2SNRP probably means snRNA:U2, a small nuclear ribonucleoprotein gene on the 2nd at 34A-B or 38A4 (personal communication from Kendal Broadie)

## Muscle tissue promoters show variable spatial distribution in larval development

**Figure 2.**
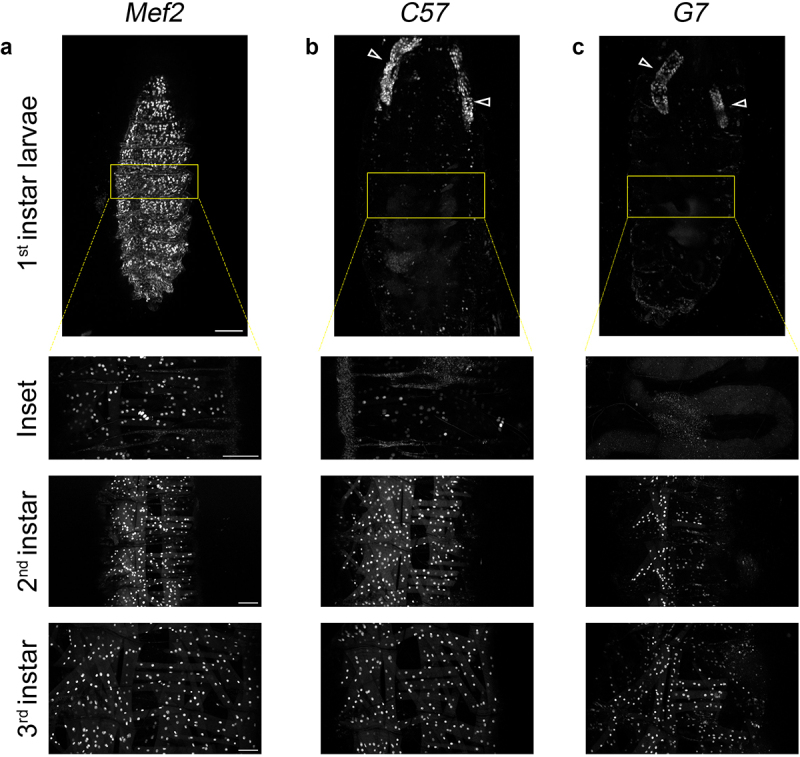
Variable spatial and temporal distribution of mCh under different muscle tissue promoters during larval development. (a-c) Maximum intensity projections of somatic muscles visualized using mCh throughout larval development. (a) *Mef2* drives expression in all somatic body wall muscles during the 1^st^, 2^nd^, and 3^rd^ instar larval stages. (b) Expression of mCh by *C57*-GAL4 is first observed in 1^st^ instar larvae and persists throughout larval development. (c) *G7*-GAL4 turns on mCh expression in 2^nd^ instar larvae and persists throughout the 3^rd^ instar larval stage. White arrows indicate salivary gland expression of *C57*-GAL4 and *G7*-GAL4. Scale bar: 50 μm.

To confirm the reported gene expression patterns for the chosen muscle-GAL4 drivers, we investigated larval development spanning from the 1^st^ to the 3^rd^ instar stages. As expected, *Mef2*-GAL4 showed strong expression of mCh in all somatic body wall muscles throughout larval development (Figure 2a, left panels). In contrast, weak expression of mCh was first observed in the body wall muscles of 1^st^ instar larvae using *C57*-GAL4 (Figure 2b, middle panels) and not observed until the 2^nd^ instar larval stage with *G7*-GAL4 (Figure 2c, right panels). Though enriched in somatic muscles, the *C57* and *G7* promoters also expressed mCh in salivary glands (white arrowheads). Together, these results confirm that each GAL4 driver shows a unique pattern of spatial and temporal target gene expression. To our knowledge, this salivary gland expression has not been reported and if not taken into account, could confound studies involving intertissue communication.

## Comparison of mCh protein expression levels throughout *Drosophila* development

**Figure 3.**
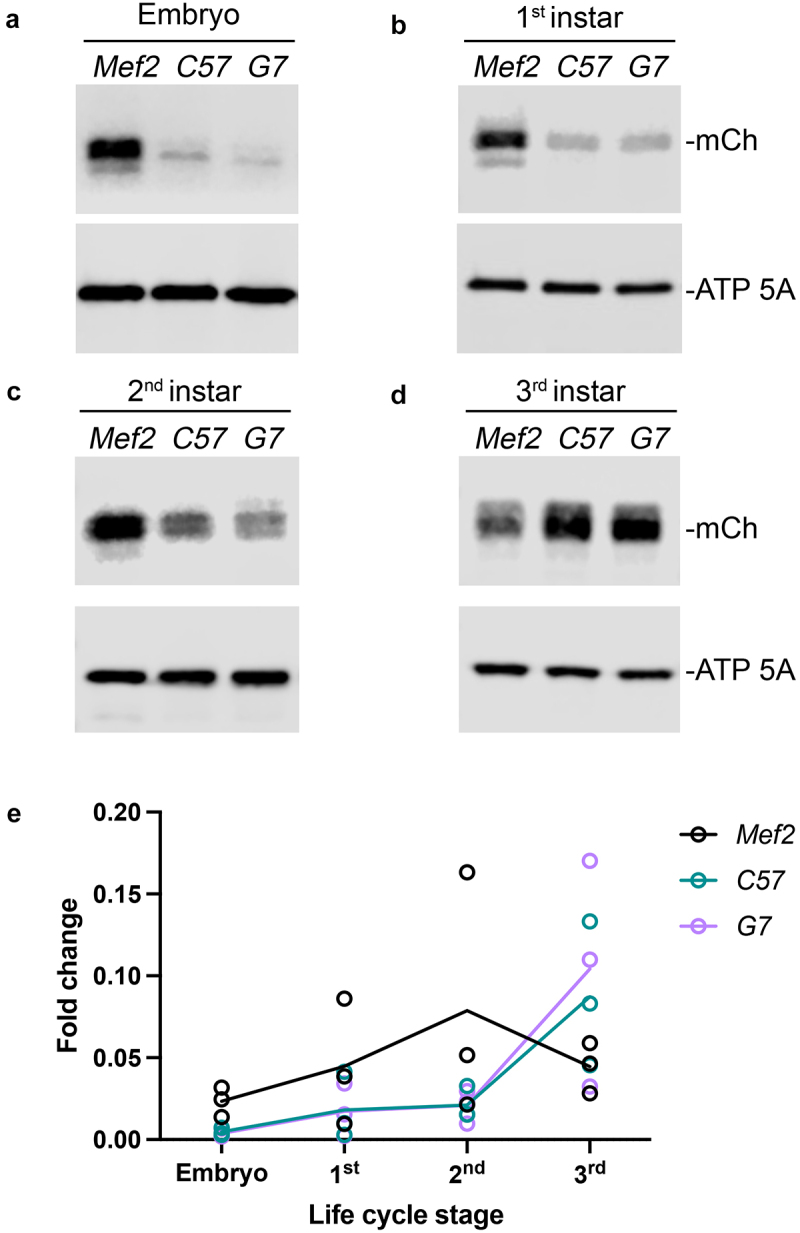
Western blot of mCh under control of muscle GAL4 drivers. (a-d) Western blot analysis of protein expression in the indicated stages of *Drosophila* development using mCh as a reporter. ATP5A is used as a loading control. (e) Quantification of mCh protein levels throughout development. *N* ≥ 3.

We next employed Western blotting to determine protein expression levels of mCh throughout embryonic and larval muscle development. During myogenesis, only the *Mef2*-GAL4 driver produced strong mCh signal, consistent with our confocal microscopy images (Figure 3a). Weak mCh expression was evident in 1^st^ instar larvae using *C57*-GAL4 and *G7*-GAL4 (Figure 3b). Expression in the salivary glands likely accounts for some of this signal. 2^nd^ instar larvae showed higher levels of mCh protein under control of the *C57* and *G7* promoters, albeit still lower compared to the *Mef2*-GAL4 promoter (Figure 3c). In the 3^rd^ instar larvae stage, *C57*-GAL4 and *G7*-GAL4 both express more of the mCh target protein than the *Mef2*-GAL4 driver (Figure 3d). Quantitation of GAL4 driver strength over time shows that *Mef2*-GAL4 expression was high throughout the embryonic and larval stages and decreased in 3^rd^ instar larvae compared to *C57*-GAL4 or *G7*-GAL4 (Figure 3e).

**Figure 4.**
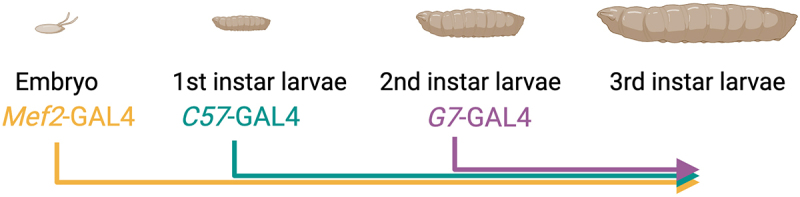
Summary of muscle GAL4 driver expression throughout *Drosophila* development. The *Mef2*-GAL4 driver begins to express target genes in mid-embryogenesis throughout the lifespan of *Drosophila*. *C57*-GAL4 and *G7*-GAL4 turn on expression at the 1^st^ or 2^nd^ instar larval stages, respectively.

A comprehensive and precise understanding of temporal and spatial expression patterns is informative for selecting the most appropriate muscle GAL4 driver in answering specific biological questions. Using two independent methods, our results confirm that *C57*-GAL4 expression is initiated in 1^st^ instar larvae, while *G7*-GAL4 induction begins in 2^nd^ instar larvae (Figure 4 and Table 1). Based upon these results, the *C57* and *G7* drivers are useful for understanding how gene manipulation post-embryogenesis affects muscle structure, function, and/or growth. Importantly, these larval muscle promoters can be used to bypass embryonic lethality resulting from the use of *Mef2*-GAL4. Altering gene expression after the completion of embryonic sarcomerogenesis also allows for distinguishing between sarcomere formation or sarcomere addition during larval muscle growth. Finally, the use of *G7*-GAL4 in 2^nd^ instar larva also eliminates using dual expression systems such as GAL4/GAL80^ts^ or drug-inducible gene expression whereby temperature or drug toxicity may show leaky expression or behavioural changes [14].

## Data Availability

Data sharing is not applicable to this article as no new data were created or analysed in this study.
